# Notes on psychiatrist liability around the world regarding suicide

**DOI:** 10.3389/fpsyt.2024.1466325

**Published:** 2025-01-23

**Authors:** Anna Saya, Giuseppe Albanesi, Diego Cichetti, Matteo Di Molfetta, Yuri Guarino, Cinzia Niolu, Alberto Siracusano

**Affiliations:** ^1^ Chair of Psychiatry, Department of Systems Medicine, University of Rome Tor Vergata, Rome, Italy; ^2^ Psychiatry and Clinical Psychology Unit, Department of Neurosciences, Fondazione Policlinico Tor Vergata, Rome, Italy

**Keywords:** suicide, claims, civil and criminal psychiatrist liability, self-determination, guarantee position, hospitalization, mental health legislation, ethics

## Abstract

**Background:**

Currently, there is a lively debate regarding involuntary treatment and the psychiatrist's liability for suicide of patients with serious mental illness. This article aims to highlight the worldwide differences, considering that in some countries suicide is still considered a crime, while elsewhere, access to euthanasia/medically assisted suicide is allowed even for psychiatric patients.

**Methods:**

Data have been collected from accredited sites, governmental websites, and databases of organizations. The legislation and socio-cultural contexts of different countries are considered.

**Results:**

This article highlight significant legislative differences, including suicide prevention programs, also due to several sociocultural patterns. The psychiatrist liability is not always clearly described in the legislation of different countries.

**Conclusions:**

What emerges from this study is the gray area of psychiatric patient suicide. Is it possible to make the psychiatrist liable for an unmanageable illness? What are the correct guidelines? When the possibility of coercion is no longer valid to avoid suicide and when does the right to self-determination begin for the psychiatric patient?

## 1 Introduction

The subject of this article is the study of international guidelines on psychiatrist's liability in case of patient's suicide, giving a portrait of the actual law profile of some states around the world. Our goal is to reflect on the role of the psychiatrists in the prevention and management of patients at risk of suicide, correlating it with the legal aspects of individual states globally. The question of institution or healthcare team liability is increasingly raised (94). The World Health Organization (WHO) claims that every year more than 700,000 people commit suicide (117). For each one, there are many more attempted (about 14 million) that are considered the most important risk factor for future suicide. In low and middle-income countries 77% of suicides occur (117).

Psychiatrist's civil and criminal liability coincides with their position of guarantee towards the patient and the community. The term position of guarantee refers to the obligations of the professional that society deems necessary as the social guarantee that he must provide through his work. This element refers to various legal foundations, the first of which is Article 32 of the Italian Constitution which establishes health as a fundamental right of the individual but also as an interest of the community. The causal relationship establishes that not preventing an event is equivalent to causing it. The medical-legal issue concerns the causal link with specific reference to predictability and preventability (23). The International Covenant on Economic, Social and Cultural Rights (ICESCR), a United Nations treaty (1976), establishes three obligatory duties for countries about the right to healthcare and human rights: to respect, protect, and fulfill the right to healthcare (53). In fact, for cultural and for some scientific prospective (developed tools to quantify suicide risk, with modest results), suicide is increasingly interpreted as the expression of a psychiatric disorder and is therefore, at least theoretically, diagnosable and preventable. The difficulty in recognizing the will of suicidal acts beyond any reasonable doubt, and even more to demonstrate the opposite, make it nearly impossible to establish liability. The need to rationally explain an attempted suicide, itself often full of contradictions, is a need as much the clinicians narcisistic frustration as for the legal concerns (15).

Some states (including Belgium, Holland, Spain, and Canada) consider suicide, in specific cases, as an act of self-determination. This idea forms the theoretical basis for euthanasia for psychiatric patients (101). For example, in Canadian law, suicide can be a carefully considered choice and the right to commit suicide includes also the right to non-interference by others. From a libertarian perspective of suicide, the duty of care is proportionate to the needs of the patient, depending on their mental state. Thus, the focus is on the possibility that suicide arises from psychological pain and not from a diagnosed mental disorder and can be a voluntary decision taken after evaluating the benefits, risks and consequences. The equation between mental illness and irrationality is criticized in the libertarian perspective as there could be a limited irrationality and, sometimes, suicide would be the result of a freely taken decision (49). Out of 192 independent states, 25 consider attempted suicide to be a crime and another 20 follow Islamic law or Sharia law, where attempted suicide can also be punished. Where attempted suicide has been decriminalized, there is no evidence that it has led to an increase in attempts. Additionally, aiding suicide has also been decriminalized in Canada and according to the legislation there is no interference with people who refuse life-saving treatment in favor of the right to decide about their own future (67). This article aims to highlight the differences and the evolution of the psychiatrist's liability worldwide. An interesting element is the significant gap between legislation and reality regarding mental illness and suicide in various countries (especially in Africa and Asia). Another aspect that emerges is the partial contradiction between the legislation and its application (e.g. Italy).

It is important to underline that some of the states are missing a clear statement of rules, laws and guidelines regarding this subject: the different criteria of possible liability are subject to very heterogeneous procedural systems and that no generalisation can actually be deduced.

## 2 Methods

The articles have been chosen, both as regards historical citations and current procedures, by searching on accredited sites (e.g. PubMed, Google Scholar, and MedLine), governmental internet sites, databases of organizations (e.g. WHO MiNDBank). The most recent search was conducted on 24 May 2024. Out of a total of 208 publications consulted, we selected 119 articles for our bibliography, researching, in addition to scientific articles, the legislative sources specific to each mentioned country. The keywords we used include suicide behavior, suicide criminalization, suicide epidemiology, suicide prevention, suicide risk, attempted suicide, self-determination, mental health law, civil and criminal psychiatric liability, psychiatric malpractice suits, litigation, claims, involuntary treatment, euthanasia, ethics, culture, spirituality, depression, social support, human rights, gender, psychiatric disorders, hospitalization, guarantee position, countries considered. For the historical part, we have used legislation and articles from 1904 to the most recent available. Regarding the results, the articles were selected from publications from 1977 to 2024. We used mainly English language articles with high bibliographic sources that were published in international literature. Whenever possible, we included the most recent bibliographies.

The results are presented in alphabetical order, divided by geographical region (**Table 1**).

**Table 1:** Data extraction.

REF.	First Author/Year	COUNTRY	TYPE	CATEGORY	SEARCH ENGINE	KEYWORDS
(1)	Adewuya 2020	Nigeria	Statistics	Suicide	PubMed	suicide, suicidal behaviors, Africa, Nigeria, adolescents
(2)	Adinkrah 2016	USA	Review	Legislation	Research Gate	legislation, anti-suicide, Africa, prevention
(3)	Ahn (KW) 2007	Korea	Opinion	legislation, culture/social norms	Google Scholar	Korea, mental health law
(4)	Ahn (SI) 2020	Korea	Statics	legislation, malpractice	Google Scholar	Korea, mental health law, malpractice
(5)	Alexandrov 2021	Netherlands	Opinion	legislation	Pubmed	mental health legislation,Netherlands
(6)	Appel 2019	USA	Opinion	legislation, ethics, malpractice	Pubmed	psychiatric liability, ethics
(7)	Appelbaum 2007	USA	Opinion	Ethics, legislation	Pubmed	Psychiatric liability, ethics
(8)	Appelbaum 2007	USA	Essay	Legislation, malpractice, ethics	Research Gate	Psychiatric liability, suicide prevention
(9)	Ariani 2023	Indonesia	Opinion	ethics, culture/social norms	Google Scholar	suicide prevention, Indonesia
(10)	ASEAN Secretariat 2016	Thailand	Essay	legislation,cultural/social norms, epidemiology	Google	Thailand, mental health
(11)	Bastiampillai 2017	USA	Review	Prevention, legislation	Research Gate	Suicide prevention, psychiatry liability
(12)	Baldaçara 2022	Brazil	Systematic review	epidemiology, suicide	Google Scholar	epidemiology, suicide, Brazil
(13)	Baquedano 2011	Mexico	Opinion	cultural,suicide,social norms	Google (Oxford academic)	Maya traditions, Mexico, suicide
(14)	Beauchamp 1993	World	Book	Cultural, ethics,	Google	Suicide, ethic
(15)	Biondi 2016	Italy	Opinion	legislation, ethics	Pubmed	suicide risk, law
(16)	Bleich 2011	Israel	Opinion	legislation, defensive medicine	Pubmed	Israel, suicide, mental health
(17)	Borges 2019	Mexico	Review	epidemiology,suicide, cultural norms	Google Scholar	suicide ideation, suicide behaviour, Mexico
(18)	Brandão 2015	Brazil	Opinion	suicide/cultural/social norms	Google	Brandão 2015, comportamento suicida
(19)	Brandt-Christensen 2012	Denmark	Review	legislation	Pubmed	Denmark, mental health law
(20)	Cabinet 2017	Japan	Law	legislation	WHO Mindbank	Japan
(21)	Chen (HH) 2012	China	Law	Legislation	Pubmed	mental health law, China
(22)	Chen (YY) 2012	China	Opinion	cultural/social norms	Pubmed	prevention suicide, Asia
(23)	Civil Code	China	Law	Legislation	Google	China, Civil Code
(24)	Coluccia 2017	Italy	Review	legislation, prevention	Pubmed	liability psychiatrist
(25)	Consiglio Federale 2011	Switzerland	Law	Legislation, assisted suicide	Government site	Assistenza al suicidio, responsabilità psichiatrica
(26)	Court of Queen's Bench of Alberta 1991	Canada	Law	legislation	Google Scholar	Psychiatrist liability, Canada
(27)	Cupelli 2016	Italy	Opinion	legislation	Google	responsabilità psichiatra
(28)	Datta 2016	Ireland	Review	legislation	PubMed	Mental health law, involuntary detection
(29)	De Hert 2022	Belgium	Opinion	legislation	Pubmed	euthanasia, psychiatric disorders
(30)	Deshpande 2013	India	Opinion	legislation, culture	Research Gate	legislation, mental health, Egypt, India
(31)	Doernberg 2016	Netherlands	Essay	legislation	Pubmed	euthanasia, psychiatric disorder
(32)	Douzenis 2014	Greece	Essay	legislation	Pubmed	mental health, law, Greece
(33)	Drew 2013	UK	Review	human rights, legislation	Research Gate	legislation, mental health, human rights, Africa
(34)	Duncan 2019	New Zealand	Opinion	legislation	Research Gate	Psychiatrist liability, suicide, NEw Zealand
(35)	Egyptian Government 2009	Egypt	Law	legislation	Government site	Mental health law, Egypt
(36)	Elliott 2001	Nevada	Review	social norms	Research Gate	gender, depression, stress, social support
(37)	Elnemais 2017	Egypt	Opinion	culture/social norms, legislation	Research Gate	Egypt, mental health law
(38)	Freckelton 2017	Australia	Review	legislation	PubMed	Psychiatrist liability, suicide, Australia
(39)	French Government 2000	France	Law	legislation	Government site	Psychiatric liability,
(40)	Ghanaian Government 2012	Ghana	Law	legislation	-Government site	Ghana,mental health law
(41)	Government of Argentina 2010	Argentina	Law	legislation	Who Mindbank	Ley 26.657, Argentina, suicide
(42)	Government of Argentina 2015	Argentina	Law	legislation	Who Mindbank	Ley 27.130, Argentina, suicide
(43)	Gunnell 2008	Mexico	Review	human rights, legislation	PubMed	governance, mental health services, mental health policy, primary care
(44)	Hanlon 2019	UK, Ethiopia, South Africa	Review	culture/social norms, legislation	Pub Med	Ethiopia; financial coverage; mental disorders; mental health; Sub-Saharan Africa; universal health coverage.
(45)	Hanlon 2017	Ethiopia, UK, South Africa	Qualitative study	social norms	PubMed	governance, Ethiopia, mental health services, mental health policy, primary care
(46)	Hauber 2019	USA	Observational study	Suicide, cultural/social norms, legislation	PubMed	Psychiatrist liability, suicide, U.S.A.
(47)	Helal 2022	India	Review	legislation	Google Scholar	suicide law, India
(48)	Hellner 2001	Scandinavia (Sweden)	Opinion	legislation	PubMed	Psychiatrist liability, suicide, Sweden
(49)	Ho 2014	Canada, USA	Essay	legislation, ethics	Pubmed	suicide, ethics, responsability
(50)	Hongally 2019	India	Review	legislation	Google Scholar	India, mental health, liability
(51)	Italian Government 1904	Italy	Law	legislation	Google	Italia, legislazione, salute mentale
(52)	Italian Government 1978	Italy	Law	legislation	Google	Italia, legislazione, salute mentale
(53)	Irmansyah 2009	Indonesia	Opinion	legislation,culture/social norms	Pubmed	Indonesia, mental health law
(54)	Jacobs 2010	USA	Guidelines	Guidelines, legislation	PubMed	Psychiatrist liability, suicide, guidelines
(55)	Jørstad 2002	Scandinavia (Norway)	Review	legislation	Pubmed	Psychiatrist liability, suicide, Norway
(56)	Kahn 2013	Sweden	Essay	legislation	Research Gate	decriminalization, suicide, Ghana
(57)	Kidd 2002	Belgium	Law	legislation	Google (peer-review source)	
(58)	Kenyan Government	Kenya	Law	legislation	Government site	Kenya, mental health law
(59)	Leenaars 2002	World	Review	legislation, ethics	Google Scholar	legislation, suicidology
(60)	Lew 2022	Australia, USA, Malaysia, China Taiwan	Review	legislation	PubMed	criminalization; decriminalization; suicide.
(61)	Lorenz 2019	Germany	Opinion	legislation	Google (peer-review source)	Psychiatrist liability, suicide, Criminal liability
(62)	Lorettu 2020	Italy	Review	legislation,cultural/social Norms	Pubmed	liability, psychiatrist
(63)	Maila 2021	Zambia	Review	legislation	Research Gate	mental diisorder, mental health legislation, Mental Health Act, Zambia
(64)	Martínez 2009	Argentina	Textbook	suicide,ethics,culture	Google Scholar	suicide prevention, Argentina, suicidology
(65)	Martin-Fumadó 2015	Spain	Opinion	legislation	Pubmed	Medical professional liability
(66)	Ministry of Public Health, Guyana 2014	Guyana	Law	legislation	WHO mindbank	suicide prevention, Guyana
(67)	Mishara 2016	World	Review	legislation	Google Scholar	suicide law world
(68)	Mondal 2019	India	Essay	Epidemiology, legislation	Researchgate	Suicide,mental health law, India
(69)	Morgan 1979	USA	Book	suicide, self-harm	–	death, self-harm, suicide, euthanasia
(70)	Mostardini 2017	Austria	Essay	legislation	Google	Psychiatrist liability, european court for human right
(71)	Murty 2004	India	Cases Report	legislation	Google Scholar	suicide in hospital, liability, India
(72)	Nadarajah 2021	USA	Review	Legislation, suicide prevention	Pubmed	Psychiatrist liability, duty of care
(73)	Narayan 2013	India	Review	legislation	Google Scholar	mental health law, India
(74)	Naveed 2017	Pakistan	Review	legislation, psychiatry	Pubmed	suicide, Pakistan
(75)	Nicolini 2022	Belgium, Netherlands	Essay	legislation, epidemiology,culture/social norms	Pubmed	euthanasia, psychiatry, Netherlands
(76)	Nigerian Government	Nigeria	Law	legislation	Government site	Nigeria, mental health law
(77)	On'okoko 2010	Canada, Congo	Opinion	legislation	PubMed	mental health, legislation, Congo
(78)	Ooi 2019	Asia, NZ	Opinion	legislation	Pubmed	liability, malpractice, Asia
(79)	Osafo 2012	Ghana	Opinion	suicide, prevention	PubMed	Ghana, suicide, suicide prevention, nurse
(80)	Páez 2022	Argentina	Opinion	Suicide, legislations	Google Scholars	Suicide legal frameworks, suicide ethical aspects
(81)	Panesar 2022	World	Systematic Review	suicide, prevention,guidelines	PubMed	suicide prevention, guidelines, suicide mortality rates
(82)	Participation 1998	United kingdom	Law	legislation	Government site	Human Right, health law
(83)	Pinals 2019	USA	Review	Legislation, ethics	PubMed	Psychiatrist liability, suicide, malpractice
(84)	Pradeep 2013	Asia	Review	legislation, prevention, ethics	Google Scholar	suicide, prevention, legal, ethical, aspects
(85)	Presidency of the Council of Ministers 2022	Portugal	Law	legislation	Government site	
(86)	Presidency of the Council of Ministers 2023	Portugal	Law	legislation	Government site	
(87)	President of the Republic of Indonesia 2014	Indonesia	Law	legislation	WHOmindbank	Indonesia
(88)	Rajs 1992	Sweden	Statistics	suicide	PubMed	HIV infection: medicolegal autopsy, cause of death, suicide
(89)	Ranta 1984	Scandinavia (Finland)	Review	legislation	PubMed	Psychiatrist liability, suicide, Finland
(90)	Reiter-Theil 2018	Switzerland	Opinion	Assisted suicide	Pubmed	Assiste suicide, suicide prevention
(91)	Reyes-Foster 2023	Mexico	Opinion	legislation, cultural/social norms	Pubmed	Human Right, health law
(92)	Reuveni 2017	Israel	Cross-sectional survey	Defensive medicine	Pubmed	Defensive medicine, Israel
(93)	Royal College of Psychiatrists 2020	United Kingdom	Retrospective study	Legislation, defensive medicine	Government site	Psychiatrist liability, suicide prevention
(94)	Sabe 2020	Switzerland	Cases report	legal medicine	Pubmed	suicide, psychiatry
(95)	Savani 2020	Asia	Review	cultural/social norms	Google	suicide, Asia
(96)	Saya 2019	World	Review	legislation, cultural/social norms	Pubmed	Involuntary treatment, psychiatry
(97)	Schott 1987	Ghana	Opinion	traditional law, religion	Google	African religions, Ghana, ethics
(98)	Sher 2015	USA	Review	legislation	PubMed	Psychiatrist liability, suicide, USA
(99)	Shinfuku 2016	Japan	Essay	culture/social norms,legislation	Google Scholar	Japanese culture, mental health laws, suicide
(100)	CSVD	Canada	statistics	Suicide, cultural/social norms	Government Site	suicide rate, Canada
(101)	Steinert 2022	Germany	Opinion	legislation, ethics	Google Scholar	law, psychiatry
(102)	Steinø 2013	Denmark	Retrospective study	legislation, malpractice	Pubmed	psychiatric claims, Denmark
(103)	Sufrate-Sorzano 2022	Spain	Retrospective study	legislation	Pubmed	suicide prevention, professional liability
(104)	Swift 1977	Kenya	Book	Psychiatry,legislation, culture/social norms		psychiatry, mental health, Kenya, Africa
(105)	Thai Government 2008	Thailand	Law	legislation	Google	Thailand, mental health law
(106)	Ugandan Government	Uganda	Law	legislation	Government site	mental health law, Uganda
(107)	United States Centers for Disease Control and Prevention 2019	USA	Statistics	Suicide, cultural/social norms	Government site	suicide rate, U.S.A.
(108)	United Nations News 2019	USA	Opinion	culture/social norms	Google - UN.org	suicide prevention, Africa
(109)	United States Centers for Disease Control and Prevention 2019	USA	Statistics	Suicide, cultural/social norms	Government site	Suicide rate, U.S.A.
(110)	Volpi 2022	Central and south America	Review	epidemiology,culture, suicide	Google Scholar	prevention suicide, Latin America
(111)	Wada 2021	Nigeria	Opinion	culture/social norms	PubMed	mental health law, Nigeria
(112)	Walker 2017	Ghana	Review	legislation	PubMed	Ghana, legislation, mental health
(113)	Wasserman 2020	Europe	Review	legislation, ethics	Pubmed	legislation, compulsory admission, european countries
(114)	Witono 2012	Indonesia	Opinion	ethics, cultural/social norms	Google Scholar	mental health, spirituality, Indonesia
(115)	World Health Organization 2022	World	Guidelines	Guidelines	Google	Guidelines, suicide
(116)	World Health Organization MiNDBANK 2006	Japan	Law	legislation	WHOmindbank	Japan
(117)	World Health Organization 2021	World	Statistics	epidemiology	Google Scholar	epidemiology, suicide, WHO
(118)	World Health Organization 2014	Argentina	Statistics	epidemiology/culture	WHO mindbank	Argentina, WHO, prevention suicide
(119)	Xu 2022	China	Review	legislation	Pubmed	mental health, China
(120)	Young 2002	Japan	Opinion	ethics, cultural/social norms	Pubmed	Japan,suicide, culture
(121)	Zaky 2009	Egypt	Essay	legislation	PubMed	Egypt, legislation, compulsory treatment, involuntary admission
(122)	Zanchetti 2004	Italy	Opinion	legislation	Google	responsabilità penale, psichiatra, suicidio
(123)	Zigmond 2017	United Kingdom	Review	legislation	Pubmed	Mental Health, UK,
(124)	Zhang 2015	World	Review	legislation	Pubmed	mental disorder, involuntary treatment

## 3 Results

### 3.1 Africa

The African region's (**Table 2**) suicide rate is the highest, 11.2 per 100,000 population in 2019, compared to the global average of 9.0 per 100,000 (60). The male suicide rate in Africa is notably high at 18 per 100,000, versus the global average of 12.4 per 100,000 (60).

**Table 2:** Africa.

COUNTRY	LAW	CARE & LIABILITY	CRIMINAL SUICIDE	SUICIDE RISK CRITERION FOR INVOLUNTARY TREATMENT	MALPRACTICE
The Democratic Republic of Congo	No specific legislation.	No specific legislation.	Suicide not criminalized.	No specific legal criteria; treatment depends on available resources and general practices.	Limited guidelines on psychiatrists' responsibilities.
Egypt	Mental Health Act (71/2009).	Psychiatrists responsible for prevention.	Suicide itself is not a crime, but involuntary admission is regulated.	Imminent danger to self and others. Requires psychiatric evaluation and court approval.	Liability if legal criteria are not followed.
Ethiopia	No specific legislation.	No specific legislation. Resource limitations affect liability.	Suicide not criminalized.	No specific criteria; relies on general guidelines.	Limited guidelines on psychiatrists' responsibilities.
Ghana	Mental Health Act (2012).	court approval for involuntary treatment, with physician responsible for patient care.	Non-fatal suicide attempts can lead to arrest, imprisonment, or fines	Imminent danger to self and others. Requires two medical recommendations and court approval.	Responsibility for ensuring compliance with legal procedures for involuntary treatment.
Kenya	Mental Health Act (2012).	Physicians' responsibility under involuntary admission.	Misdemeanor for attempts; incitement criminalized.	Imminent danger to self and others. Imminent harm requires medical assessment for involuntary admission.	Potential liability for procedural errors.
Nigeria	National Mental Health Bill (2023).	Guidelines in new legislation but very limited resources.	Misdemeanor for attempts; incitement criminalized.	Imminent danger to self and others. Requires two medical recommendations and court approval.	Psychiatrists' responsibilities are influenced by resource limitations and socio-cultural factors.
Uganda	Mental Health Act (2018).	Significant responsibility for psychiatrists under the Mental Health Act, mainly with procedures for involuntary admission.	Misdemeanor for attempts; incitement criminalized.	Written request and psychiatric evaluation within 48 hours.	High responsibility linked to patient safety.
Zambia	Mental Health Act (2019).	Responsibilities in preventing suicide.	Suicide not criminalized.	Safeguards for involuntary treatment for non-consenting patients.	Potential liability for procedural errors.

Factors contributing to suicide in Africa include a significantly higher risk among HIV-positive individuals, who are 35–40 times more likely to commit suicide than HIV-negative persons (88). Suicide rates are likely under-reported due to inadequate diagnosis of psychiatric conditions (36) and cultural differences in the manifestation of depression, which often presents as somatization rather than guilt or self-reproach (69). This cultural stigma is compounded by prevalent moralistic views on suicide, even among some healthcare professionals (79).

In Ghana, for example, cultural beliefs severely stigmatize suicide, affecting community attitudes and hindering open discussion about suicidal thoughts (97, 104, 56). Criminalization of suicide attempts may lead to more lethal methods being employed, although it can also act as a deterrent in some cases. Evidence from countries like Canada and Ireland shows increased suicide rates post-decriminalization, potentially due to more accurate death reporting (56).

Decriminalizing suicide shifts the focus to understanding and mitigating its social and environmental causes rather than punishment (56). Africa's psychiatric services are hampered by severe resource constraints, with Nigeria having only 250 psychiatrists per 200 million people, far below the WHO recommendations. Government spending on mental health is critically low, averaging $0.46 per capita in 2022, compared to the WHO's recommendation of $2.00 (108).

In 2022, African health ministers set 2030 targets to enhance mental health policies, implementation, and funding (108) which will increase the role of the psychiatrist. Psychiatrists in Africa play a vital role in suicide prevention, requiring them to navigate cultural complexities, advocate for mental health awareness, and collaborate with governments and NGOs to formulate culturally sensitive mental health policies. These policies must address resource allocation, service accessibility, and public education to effectively mitigate suicide rates. It will be interesting to learn in future studies what impact these changes will have on psychiatrist liability.

#### 3.1.1 The Democratic Republic of Congo

Suicide is not legally punishable in the Democratic Republic of Congo. Mental healthcare is provided mostly by private and general hospitals, even if a national health sector plan from 1999 emphasizes the integration of mental health services into primary care through the national mental health program (77).

The country has agreed to international legal standards regarding the rights and protection of individuals with mental illnesses but lacks specific national legislation defining these rights and protections, procedures for voluntary or involuntary hospitalization in psychiatric facilities, and clearly defining psychiatrists' responsibilities for preventing and treating suicide (77).

#### 3.1.2 Egypt

In Egypt, suicide is not legally punished. The Egyptian parliament ratified the first Mental Health Act in 1944. This law regulated involuntary detention of psychotic patients, second expert opinions, consent to treatment, and appeals, forming the basis for hospital psychiatry practices for about 30 years (121). In 2006, the General Secretariat for Mental Health within Egypt's Ministry of Health initiated a review and drafting of a new Mental Health Act to revise criteria for compulsory admission to psychiatric hospitals (30).

By 2008, the final draft submitted to Parliament distinguished between compulsory admission and treatment (35). The established criteria for compulsory admission include an imminent danger to self or others, giving the psychiatrist primary responsibility for preventing suicide (37).

#### 3.1.3 Ethiopia

Ethiopia, a low-income country in sub-Saharan Africa with a population of 100 million, has few mental health professionals, with around 60 psychiatrists in the public sector, mostly in the capital, Addis Ababa. The mental health care treatment gap is high, with an estimated 90% of people with mental health conditions not receiving evidence-based care, and less than 1% receiving community-based care (45).

There is no specific legislation in Ethiopia regulating involuntary treatments for people with mental illnesses, nor laws protecting the rights of people with psychiatric conditions or at risk of suicide. The country is a signatory of the United Nations Convention on the Rights of Persons with Disabilities, which includes the rights of people with mental disabilities. Suicide is not criminally punishable in Ethiopia. The lack of adequate legislation and the scarcity of resources complicate the examination of the psychiatrist's liability regarding suicide prevention (44).

#### 3.1.4 Ghana

In Ghana, attempting suicide historically carried severe penalties such as decapitation and property confiscation, reflecting deep-rooted cultural stigma. Individuals who attempt suicide can be swiftly arrested, imprisoned for up to two years, fined heavily, or both (112). Currently, non-fatal suicidal behavior is criminalized under Ghana's penal code (Act 29, section 57, subsection 2) (2).

Ghana's Mental Health Act of 2012, endorsed by the WHO, allows for involuntary admission and treatment of severe mental disorders, requiring court approval based on medical recommendations (33). An application to court for temporary involuntary treatment requires two medical recommendations and detailed reasoning for the necessity of treatment. The treatment aims to improve the person's condition, restore decision-making capacity, minimize risks for patients and others (40), and confer responsibility for the health of the patient to the doctor.

#### 3.1.5 Kenya

Kenyan laws regarding nonfatal suicidal behavior and abetment of suicide are outlined in the Kenyan Penal Code (58). Section 225 states that inducing, counseling, or aiding another person to commit suicide is a felony punishable by imprisonment for life, while Section 226 declares that attempting suicide itself is a misdemeanor (58).

The Kenyan National Commission on Human Rights plays a central role in overseeing mental health well-being. The Act allows involuntary admission to mental health facilities if a qualified medical practitioner deems it necessary due to imminent harm to oneself or others or for treatment purposes. Under this procedure, responsibility for the patient's life falls on the attending doctor (2),

#### 3.1.6 Nigeria

In Nigeria, federal law criminalizes non-fatal suicidal behavior, allowing for arrest and prosecution (76). The penal code stipulates that attempting to kill oneself is a misdemeanor punishable by a one-year imprisonment. Additionally, incitement to suicide is criminalized, with those causing, advising, or assisting another to kill themselves liable to life imprisonment (2).

With over 190 million people, Nigeria has fewer than 300 psychiatrists, resulting in a ratio of about one psychiatrist per 700,000 people (111). Significant progress was made with the signing of the National Mental Health Act in January 2023, which represents the first comprehensive mental health legislation in Nigeria since the Lunacy Act of 1958. This act aims to improve mental healthcare and achieve guidelines for the compulsory hospitalization of patients with mental health conditions. Admissions can occur if there is a significant likelihood of imminent harm. This decision must be supported by two independent qualified physicians and is subject to review by a designated committee, re-establishing the psychiatrist's responsibility toward patients with suicidal ideation (1). However, the psychiatrists' responsibility is influenced by socio-cultural factors and limited resources.

#### 3.1.7 Uganda

Uganda criminalizes both abetment of suicide and suicide attempts (106). Attempting suicide, is considered a misdemeanor, leading to immediate arrest and prosecution (2, 33).

The 2018 Mental Health Act of Uganda establishes the Uganda Mental Health Advisory Board. It mandates mental health treatment at primary health centers and outlines procedures for involuntary examination, admission, and treatment. Involuntary admission requires a written request to the head of a mental health unit, followed by psychiatric evaluation within forty-eight hours (33). Thus, psychiatrists in Uganda bear significant responsibility for ensuring the well-being and protection of patients' lives.

#### 3.1.8 Zambia

In Zambia, attempted suicide is no longer considered a crime following the Chapter 89 of the penal code, known as The Suicide Act. Section 3 of this chapter explicitly states that suicide is no longer an offense under common law, and therefore, survivors of suicide attempts face no legal penalties (63, 2).

Previously, the Mental Health Disorders Act of 1951 in Zambia lacked protections for individuals with mental disabilities and did not provide clear guidelines for involuntary admission and treatment. However, in 2019, the enactment of the Mental Health Act introduced safeguards for involuntary admission, specifically for patients unable to provide informed consent due to their mental condition or who refuse necessary services for their own or others' protection (33). This legislation marks a significant advancement in Zambia's mental health system and clarifies the responsibilities of psychiatrists in preventing suicide.

### 3.2 The Americas

In relation to suicide prevention, the countries of Latin America, guided and accompanied by the Pan American Health Organization (PAHO), have been making progress in the construction of general mental health and suicide prevention programs (118). In 2012, when the first WHO document Public health action for the prevention of suicide was published, only Nicaragua and Panama in Central America, and Colombia and Uruguay in South America had national plans for the prevention of suicide. In 2016, The Pan American Health Organization published the document Prevention of suicidal behavior corresponding to a compilation of chapters regarding the epidemiology and experiences in the prevention of suicidal behavior from different countries in the region (110). However, the Americas (Latin America, the United States and Canada) (**Table 3**) is the second region after Europe with the greatest development of national suicide prevention programs (81).

**Table 3:** The Americas.

COUNTRY	LAW	CARE & LIABILITY	CRIMINAL SUICIDE	SUICIDE RISK CRITERION FOR INVOLUNTARY TREATMENT	MALPRACTICE
Argentina	*Ley 26.657 Ley de la salud mental.* Government of Argentina, 2010	Responsibilities in preventing suicide.	Suicide not criminalized.	Imminent danger to self and others. Requires psychiatric evaluation and court approval.	Psychiatrist's liability in case of failure to comply with suicide prevention guidelines.
Brazil	*Lei no. 13,819. Governement of Brasil, 2019.*	Responsibilities in preventing suicide.	Suicide not criminalized.	Imminent danger to self and others. Requires psychiatric evaluation and court approval.	Obligation not to leave the person alone, to remove dangerous tools, and to consider psychiatric consultancy or hospitalization.
Canada	Provincial laws: Ex. Health Professions Act (Alberta), Regulated Health 274 Professions Act (Ontario)	Responsibilities in preventing suicide (duty of care)	Suicide not criminalized. Legal Euthanasia. Euthanasia allowed for psychiatric patients.	Likelihood of significant mental or physical deterioration. (depends on the Province)	Psychiatrist's liability in case of failure to comply with suicide prevention guidelines.
Guyana	Ministry of Public Health, Guyana. *National Suicide Prevention Plan 2015–2020*. Ministry of Public Health, Guyana, 2014	The initiative to tackle the alarming phenomenon of suicide does not come so much from institutions but from non-profit organizations such as the Guyana Foundation.	The act of committing suicide, or even trying to commit suicide, is illegal and carries a two-year prison sentence.	No specific criteria; relies on general guidelines.	Psychiatrists' responsibilities are influenced by resource limitations and socio-cultural factors. Given the high frequency of the acts, the liability for suicide doesn't lie with psychiatrists (the overall suicide rate is 44.2 per 100,000).
Mexico	Ley de Salud Mental del Distrito Federal, 2011	Responsibilities in preventing suicide.	Suicide not criminalized.	Involuntary hospitalization is allowed as the last therapeutic alternative, and the involuntary admission has to be authorized by an independent authority	Psychiatrists' liability is influenced by resource limitations and socio-cultural factors.
The United States of America	Model Penal Code (art. 2.01, 2.02, 2.10), state laws	Responsibilities in preventing suicide (duty of care)	Suicide not criminalized. Euthanasia legal in some territories.	Allowed, regulated by national laws	Psychiatrist's liability in case of failure to comply with suicide prevention guidelines.

#### 3.2.1 Argentina

In Argentina, the current guidelines for emergency assistance in mental health explicitly propose three lines of approach depending on the specific situation, always trying to use less restrictive methods: a) psychosocial/psychotherapeutic approach; b) psychopharmacological approach; and c) mechanical restraint (42). National Mental Health Law No. 26,657/2010 raises respect for the dignity of care by prioritizing health and freedom, does not prohibit the restraints but limits their use (41).

The national suicide rate in Argentina is 8.2 per cent per 100,000 inhabitants, compared to the 6 and 7 per cent per 100,000 inhabitants registered at the end of the twentieth century (64). The discussion of psychiatrist negligence and liability mainly focuses on cases of suicide in psychiatric patients recently admitted to a hospital setting, where the risk of certain and imminent harm is the legal requirement for involuntary psychiatric hospitalization. In a 2011 ruling, the National Civil Court upheld the first instance judgment in a case involving a schizophrenic patient with hallucinations and delusions, who fled a public hospital and died days later in a road accident. This ruling attributed to the Government of the City of Buenos Aires the responsibility for the death of the schizophrenic patient (with repeated escapes) in a road accident, a few days after escaping from the psychiatric hospital, since there is an adequate causal link between the professional and service deficiencies that allowed escape and death by suicide (…). The fact that the hospital (…) is considered open-door is not a reason to relieve the staff of responsibility for the patient's escape, since this circumstance does not mean that patients admitted can go out and roam freely on the streets of the city. In another decision, the National Civil Appeals Court, Section E, in the case of a patient, diagnosed with schizophrenia admitted to the Hospital General de Agudos Álvarez, who escaped from the hospital and then committed suicide, ruled that the institution was responsible as it had a duty to take all necessary measures to ensure that the treatment of the patient did not contravene the law (64).

National Mental Health Law No. 26,657/2010 establishes that health-care providers must provide health coverage to people who have been victims of suicide attempts and their families, as well as to the families of suicide victims, which includes detection, monitoring and treatment according to what is established by the enforcement authority. The health team must prioritize the assistance of children and adolescents without any type of prejudice or discrimination. In the first place, it raises care as a right, referring to the right to health, and secondly, it makes suicide in children visible and prioritizes care in the most vulnerable age group and the one with the highest incidence in the last decade (80).

What cannot be maintained is that the determination to commit suicide always derives from the absence of mental health as the cause of the alteration of mental faculties: this statement opens a discussion on suicide liability which could be refuted from a strictly psychiatric point of view, admitting a different medico-legal point of view, given that not all suicides are the consequence of a mental disorder (41). In Legislative Decree 603/2013, article 1, it is stated that psychological suffering must be understood as any type of mental suffering of individuals, linked to different types of predictable or unexpected crises, as well as to situations of more prolonged suffering, including disturbances and/or diseases, as a complex process determined by multiple components, in accordance with the provisions of article 3 of Law 26657. The law highlights the fact that mental suffering is not always the result of alterations in mental faculties (64).

#### 3.2.2 Brazil

The data showed that suicide rates in Brazil range between 4.6 and 6.6 per 100,000 inhabitants, from 2000 to 2020. Thus, it is ranked as the eighth country with the most suicide cases in the world in absolute numbers. (Baldaçara). Brazil has implemented a national suicide prevention strategy (ENPS) throughout the country with the publication of Ordinance no. 1,876 (14/08/2006 of the Ministry of Health). The Manual, aimed at mental health professionals, underlines three points in prevention procedures: place, time and effectiveness of the first interview to reduce the level of the crisis that triggers the suicidal act. Some aspects must be evaluated, such as mental state, the suicide plan and social support, to carry out the risk assessment: low, medium and high risk. For each level of risk there are a series of behavioral indications: people at high risk have the means and plans to carry it out immediately. In this case, the mental health professional has the obligation not to leave the person alone, to remove dangerous tools, and to consider psychiatric consultancy or hospitalization (18).

#### 3.2.3 Canada

Even in Canada, suicide is an extremely frequent cause of death, with an average of 4,500 suicides every year, a rate approximately comparable, albeit slightly lower, to that of the USA (100). Just as in the USA, provincial laws, for example the Health Professions Act (Alberta) or the Regulated Health Professions Act (Ontario) regulate the liability of the doctor and the obligations they have towards the patient, both in a civil and criminal sense. In general, however, the responsibility for proving negligence or violation of standards of care lies with the plaintiff. The case of Wenden v. Trikha (Quebec, 1991) is emblematic which, although it does not directly concern a suicide, illustrates how the responsibility of the psychiatrist and the hospital facility must be demonstrated. In this judgment, the patient, who escaped from the hospital facility where he was hospitalized on a voluntary basis, caused an accident with his car. He was convicted for the damages inflicted on the plaintiff, while both the psychiatrist on duty and the hospital were acquitted as it was not possible to prove either negligence with respect to treatment obligations, or whether these obligations persisted at the time of the escape (26).

#### 3.2.4 Guyana

In Guyana the numbers are shocking: the overall suicide rate is 44.2 per 100,000 inhabitants with an important gender distinction (men 70.8; women 22.1, WHO 2012). For each successful suicide, there are up to 25 cases of failed attempts. The percentage of suicides among Indo-Guyanese amounts to 80 percent; this demographic group represents 40 percent of the population. The act of committing suicide, or even trying to commit suicide, is still illegal and carries a two-year prison sentence. A first problem is the inability of institutions to cope or even just recognize the psychological discomforts that afflict the population, despite the widespread presence of depression, anxiety and low self-esteem. Mental health problems are still stigmatized, and neither legislation nor medical facilities can provide an adequate response, not to mention social isolation and prejudices that associate mental illness with forms of witchcraft and possession (66). The initiative to tackle the alarming phenomenon, therefore, does not come so much from institutions but from non-profit organizations. Given the high frequency of the acts, the liability for suicide doesn't lie with psychiatrists; however, the cooperation with the government is necessary to promote an adequate prevention program.

#### 3.2.5 Mexico

In Mexico the suicide rate increased from 1.13 per 100,000 inhabitants in 1970 to 6.2 per 100,000 inhabitants in 2020 (7,818 cases of suicide) an increase of approximately 275%; suicide attempts are approximately 900,000 per year (17). The suicide rate in young people between 15 and 29 years old has had a sustained increase. Suicide occupies an important place in Mayan religion and practices: in fact, the Mayans have a suicide goddess. The south-eastern region of Mexico, which geographically corresponds to the Mayan area, where the Mayan faithful lived and still live, today records the highest suicide rate in the country (13). Psychiatric liability on suicide is influenced by limited resource and socio-cultural factors.

In Mexico the suicide rate increased from 1.13 per 100,000 inhabitants in 1970 to 6.2 per 100,000 inhabitants in 2020 (7,818 cases of suicide) an increase of approximately 275%; suicide attempts are approximately 900,000 per year (17). The suicide rate in young people between 15 and 29 years old has had a sustained increase. Suicide occupies an important place in Mayan religion and practices: in fact, the Mayans have a suicide goddess. The south-eastern region of Mexico, which geographically corresponds to the Mayan area, where the Mayan faithful lived and still live, today records the highest suicide rate in the country (13). In May 2022, the Mexican Congress passed a new reform to its Ley General de Salud (General Health Law) pertaining to mental health and addiction. This new law is a progressive piece of legislation seeking to expand access to mental health services, close psychiatric hospitals, and abolish involuntary treatment. While seemingly positive, there are several significant problems inherent in the law; for example, it fails to establish appropriate infrastructure to address severe mental illness such as schizophrenia and other psychotic disorders, so people with severe psychosocial disabilities who lack a strong social support network will continue to languish in institutions where they are routinely subjected to harmful, abusive practices in violation of their human rights (91).

#### 3.2.6 The United States of America

According to the American Foundation for Suicide Prevention, in 2017 there were 1.4 million suicide attempts recorded in the United States, of which 47,174 were completed (109), making it the second leading cause of death in the 15-34 age group, fourth in the 35-54 range and among the top 10 overall (107). In the USA, as in Canada, the burden of proving an individual's responsibility for suicide is on the plaintiff (83, 72); this consists of one of the 4 Ds: duty, dereliction, damages, or direct responsibility (8, 98). The last two reasons normally apply to cases in which an individual's suicide was instigated or directly caused by the actions of another individual. However, it should be remembered that in the United States legal procedures depend on both federal laws and relevant state laws. Both in the cases of Commonwealth v. Pierce (Massachusetts) and People v. Samuel (California) psychiatrists were convicted of gross negligence towards patients whose anti-conservative will was foreseeable. The general responsibilities are however established by the Model Penal Code, in articles 2.01 (omission), 2.02 (imprudence and negligence), and 210 (manslaughter).

What concerns the doctor, and particularly the psychiatrist, are the first two Ds; in this case, a specific type of relationship exists between doctor and patient which implies the obligation of the former to pursue the well-being of the latter and to avoid their death. Negligence and errors in complying with this duty are, consequently, configured as medical liability for the patient's suicide (83). The psychiatrist's first task, according to the American Psychological Association (APA) guidelines (54), is to accurately evaluate the suicidal risk: specifically, how high it is and what the factors are that can increase (and possibly decrease) this risk. To do this, an accurate medical history is necessary, which considers possible precedents, differentiating self-harming behavior from suicidal behavior (46), a correct diagnosis, and the possible use of risk assessment scales. The psychiatrist will then have to establish the most appropriate measures to keep the patient safe. The guidelines refer to the following factors: a suitable environment and adequate supervision (in the case of hospitalized patients), a good therapeutic alliance, collaboration with the other specialists treating the patient, assessment of adherence and response to treatments, and education of both the patient and the family. It should be noted that the no harm contract, although it represents a very common practice, is not to be considered protection in the case of an incorrect assessment of the suicide risk (54). Last, but not least, is the choice of psychopharmacological treatment: the use of drugs such as Lithium and Clozapine which have shown a reduction in the suicide rate (11) is encouraged although antidepressants are to be used with caution, especially in adolescents and children, due to the possible increase in suicidal risk (11).

### 3.3 Asia

Although 60% of reported suicides worldwide occur in Asia (**Table 4**), we should consider that there are few South Asian countries in which suicide is not a crime, namely Indonesia, the Maldives, Sri Lanka and Thailand, and there may be serious under-reporting in countries where suicide is a crime. In Europe, death by suicide can only be certified by doctors, while in most Asian countries it must be certified by the judicial authority (47). In fact, while in high-income countries in the West, 90% of suicides are related to mental disorders, in Asian countries where collectivist societies prevail, suicide is considered a social more than individual phenomenon and is correlated to socio-cultural and environmental factors which, at times, cause acute stressors. An interesting study in Tajikistan shows the suicide rate is higher among female victims of violence who seek help as they are then more subject to isolation and victimization (95).

**Table 4:** Asia.

COUNTRY	LAW	CARE & LIABILITY	CRIMINAL SUICIDE	SUICIDE RISK CRITERION FOR INVOLUNTARY TREATMENT	MALPRACTICE
China	Mental Health Law of the People's Republic of China, 2012	Responsabilities in prevention by guardians who, among other things, have decision-making power for involuntary treatment for suicide risk.Psychiatrists(small percentage) as the vast majority of mental disorders are cared of general or traditional doctors	Suicide not criminalized	Substantial difference between the imminent danger to self and others.In the first case hospitalization can be imposed only if the guardians agree.	Psychiatrists responsabilities are influenced by the resource limitation and socio-cultural factors,first of all, the preeminent role of the family.Very rare claims.
India	Mental Health Care Act (2017)	Psychiatrists responsible for prevention diluited by the role of the family and the scarcity of resources	Controversial Situation:suicide criminalized under Indian Penal Code;decriminalization of suicide, under MHCA.	Imminent danger to self and others	Potential liability of psychiatrists. Very law claims.
Indonesia	Undang-Undang 2014	Care by family and psychiatrists (Abuses are widespread).Theoretical more than effective liability of psychiatrists	Suicide not criminalized	No real legal basis for involuntary treatment	Potential liability of psychiatrists.Very rare claims
Israel	The Equal Rights of Persons with Disabiities Law 578-1998?	Responsibilities in prevention by psychiatrists	Suicide not criminalized	Imminent danger to self or others if there is a causal relationship between the illness and the risk	Psychiatrist liability. Frequent claims
Japan	The Law Related to Mental Health and Welfare of Persons with Mental Disorders (2006)	Responsibilities in prevention by psychiatrists and of non-professional figures (especially the family)	Suicide not criminalized	Imminent danger to self or others	
Korea	Mental health Act 2014	Responsibilities in prevention by family and psychiatrists	Suicide not criminalized	Hospitalization necessary for the health or safety of the patient or the others	Lawsuits against psychiatrists mainly concern hospitalized patients, rarely outpatients
Pakistan	Mental Health Ordinance 2001	Responsibilities in prevention by family and potential responsibilities by psychiatrists (very limited availability of treatment)	Suicide criminalized	Prevention violent conduct of the patient towards themselves or others	Potential liability of psychiatrists.Very rare claims.
Thailand	Thai Government. Thai Mental Health Act, B.E 2551 (2008).	Potential responsibilities by psychiatrists (very limited availibility of treatment)	Suicide not criminalized	Prevention harm themselves or others	Potential liability of psychiatrists.Very rare claims

In the general context of a lower correlation between suicide and mental disorder there is a wide modulation: namely 97-100% in Taiwan and 96% in Pakistan while the lowest percentages are in China at 45-76% and in India at 33.6-88%. Regarding psychological autopsies, the percentage of depression or other disorders is much lower in Asia: e.g. in India it is 25% and in some rural areas it is as low as 2% (22).

The Asian continent modulate the psychiatrist's liability regarding suicide in a heterogeneous way essentially for the following three reasons. Firstly, the preeminent role of the family and society compared to the West (especially in collectivist societies) which, although it cannot replace the specific skills of the psychiatrist, in fact relegates the psychiatrist's role and power to a marginal level. Secondly, the importance of the religious component with the equivalence between suicide and sin with the consequence of a greater, if not almost exclusive, responsibility of the person who commits suicide, sometimes extends also to family members with the consequent imputability of those who committed or attempted suicide.

The Asian ideology identify suicide as rational or even in a self-sacrificial sense, in no way connected to a mental disorder. This last reading has been present in Japan for a long time and still constitutes a strong afterthought in Japanese culture despite adherence to Westernized legislation.

#### 3.3.1 China

The Mental-Health legislation of China became effective on 1 May 2013, reformulating the WHO's key principles (124). Articles 21 and 28 explicitly insists on the active role of the family in the management and care of people with mental disorders (21). Article 49 explains the role of the guardians in the care of non-hospitalized patients, specifying the bodies responsible for providing the patients with the necessary help. This duty is underlined by the rules sanctioning guardians who fail to fulfill their responsibilities. Article 69 underlines the broad reading of the term relatives, and support from relatives includes all types of support to persons with a mental disorder from relatives, regardless of the existence of a generational or biological relationship (21).

Regarding the dangerousness criterion in the criteria for compulsory treatment for a severe mental disorder, the substantial difference between the risk of suicide and acts against third parties appears interesting. In the first case, hospitalization can be imposed only if the guardians agree and, in case of refusal, they assume responsibility for the patient who remains at home. According to Articles 30, 31, and 32, regarding the risk of aggression, the guardians can request a review of the medical indication but do not have decision-making power as such power belongs to the two psychiatrists who have ordered it (21). These provisions would seem to limit the liability of psychiatrists in cases of suicide but not in cases of homicide similarly to China has a relatively permissive cultural tendency regarding suicide.

Another element that dilutes the liability of the psychiatrist in China is given by the observation that well over 50% of patients treated in emergency departments for attempted suicide are not diagnosed with a mental disorder (21). Due to the huge lack of services, only 7% who had made a suicide attempt had then consulted a mental health professional (84). Despite enormous changes in mental health care in China recently, a small percentage (4.5% diagnosed axis 1) sought help from mental health professionals between 2013 and 2015 while the vast majority were either not seen by a professional or were seen by doctors of general or traditional Chinese medicine, both because of the scarcity of resources and for the dominant role of the family which often keeps mental illness secret, due to the stigma (119).

However, it must be considered that the Article 1218 of the 2020 Civil Code, which provides that if a patient suffers damage in the course of diagnosis and treatment activities, and the medical institution or its medical staff is in fault, the medical institution shall bear the liability for compensation constitutes a significant reference to the psychiatric responsability also for patient suicide (23).

#### 3.3.2 India

The Mental Healthcare Act (MHCA) has been in force in India since 2017. This legislation distances itself to the point of opposing the traditional conception of mental health: if on the one hand it was considered revolutionary, on the other it was seen as an obstacle to the adequate care of patients and therefore strongly criticized (50). The most important difference is that the MHCA establishes the centrality of patients and psychiatrists while traditional conceptions consider, above all, the role of the family, and partly that of society, as an important resource in the management of mental illness. The MHCA emphasizes choosing the least restrictive alternative which considers the need for treatment and limits restrictions on the person's rights (50). The criterion for involuntary treatment includes the dangerousness criterion for themselves and others (96). There are few suicide prevention strategies at the state level in India (68).

Paradoxically, despite the MHCA, attempted suicide is still treated as an offense under section 309 of the Indian Penal Code, and until recently, there were still legal consequences regarding the treatment of the dead body or the fate of the person's or family members' property. Even if suicide will probably no longer be a crime in the near future, as deemed by the Supreme Court and the controversy over its unconstitutionality, it will still be considered deviant behavior in society and the acts and omissions connected to suicide as harm to third parties would remain a crime. Article 21 of the Indian Constitution states that no one shall be deprived of his personal liberty except in accordance with the procedures of law. The question is whether the right to live includes the right to die (47). Judicial rulings have sometimes moved towards including the right to die, and sometimes they have gone in the opposite direction with the criticism that the right to life could not be fulfilled if there is also the right to a dignified death (47). In 2008 it became mandatory to conform disability legislation to the United Nations Convention on the Rights of Persons with Disabilities (UNCRPD) with the new paradigm based on the presumption of legal capacity, equality and dignity (73), but this is not covered in the MHCA which presumes serious mental pathology, unless otherwise proven, for anyone who attempts suicide. However, the decriminalization of suicide, albeit partial and ambiguous, has favored treatment given that 93% of attempted suicides were by people with mental disorders. Despite this, both the fact that the hospital must inform the police, and the stigma associated with suicide catalyze the focus more on medico-legal aspects than therapeutic ones (68). Furthermore, services are often not available and attempted suicides are only occasionally seen by psychiatrists (84).

The figure of the psychiatrist takes on less importance than in the West and responsibility for mental illness is diluted between various figures. Until 1990, the number of malpractice cases against psychiatrists in India was very low compared to those in other specialties and the psychiatrist was often acquitted. Recently, even if there has been an increase in malpractice cases and convictions of psychiatrists around the world, suicide represents a very rare reason for legal battle in India and it is mostly limited to hospitalized patients (84). However, there is a rather narrow scope of liability for the hospital limited to only when the patient is under the complete care and protection of the hospital staff. In contrast, when the responsibility is divided between the hospital staff and the relatives, it is difficult to decide on the attribution of blame (71).

#### 3.3.3 Indonesia

Indonesian legislation has ratified international conventions and provides an adequate legal framework for the protection of human rights. The Undang-Undang of 2014 states that the government, regional government, and society must guarantee efforts in prevention, promotion, treatment, and rehabilitation, as well as accessibility to treatment. The determination of the legal competence of a person with suspected mental problems is delegated to doctors who are experts in this field and the mental state examination must be carried out by specialists (87). Unfortunately, there is a large gap between legislation and reality. Despite recent regulations, abuses are widespread, and Indonesia has long been criticized by the National Human Rights Commission. There is a strong lack of services which, in addition to hindering treatment, blocks accessibility to data, generating the impression that the mental health problem does not exist. To date, custodial treatments predominate, and unnecessary involuntary treatment is frequent. A person can be taken to hospital without their consent by anyone (husband, wife, guardian, or another family member, etc.] who feels uncomfortable with their behavior. The recent decreased availability of caregivers (family), together with the stigma also amplified by religions (Muslim, Islamic, and Hinduism - even if some suicides are considered altruistic by this religion) significantly reduces the search for help, with a consequent increase in suicides (9).

Indonesian institutions carry out pasung in which patients are often tied up, isolated, confined in small places, and with their legs in wooden stocks. A program to eliminate pasung has recently been launched (53).

The inclusion of spiritual aspects in mental health care in this country, largely linked to Islam, is hailed as desirable and almost read as a wind of modernity in the approach to mental health (114). In this context, the role and consequent liability of the psychiatrist, especially about suicide, is extremely limited.

#### 3.3.4 Israel

Failure to prevent patient suicide is a common basis for malpractice suits, and as such, increases the occurrence of the practice of defensive medicine by psychiatrists (16). In Israel, 20,6% of psychiatrists are directly subject to malpractice claims. A study states that in a questionnaire administered to 213 psychiatrists, 62.1% admitted to the use of defensive medicine. The questionnaire referred to 4 defensive medicine scenarios, including the treatment of patients at risk of suicide. In this case, defensive medicine consisted of referrals for hospitalization, even when unnecessary (92). Paradoxically, unnecessary hospitalizations can then lead to charges of improper detention, one of the most frequent reasons for lawsuits in this field. Therefore, defensive medicine does not always protect from legal liability, and more importantly, unnecessary hospitalizations can be detrimental to care. The authors highlight the frequent difficulty in predicting suicide (half of the patients do not declare their intent, or plans to implement them, to anyone) and the risks of the use of defensive medicine (92).

Limitations of the psychiatrist's liability regarding dangerousness are defined by Israeli law: for the individual who is mentally ill, who is ill and as a result of his illness his capacity for judgment or reality testing is significantly impaired […] and if the patient may endanger himself or another, with imminent physical danger, and there is a causal relationship between the illness and the risk, the psychiatrist is authorized to issue a written warrant for urgent hospitalization (16). The significant deterioration of judgment is unclear, and it is necessary to analyze the circumstances of each case (16).

#### 3.3.5 Japan

Only since 2000 has there been a greater definition of Psychiatric Social Workers (PSW) while the previous legislation included mental illnesses within the scope of disabilities together with physical and developmental ones and favored the development of non-psychiatric professional figures (99).

In Japan there are two types of involuntary treatment, the first of which requires the presence of a mental disorder and the risk of harm to oneself or others, while the second regards Admission for care and protection. Art. 27 of the Law Related to Mental Health and Welfare of the Persons with Mental Disorders regarding the protection and responsibility of people with mental disorders, explicitly highlights the active role of non-professional figures, especially the family, which traditionally has the duty to bear the burden of caring for sick members (116). It emphasizes the need for assistance and support from the institutions, not only for the patients, but also for the family and friends in their roles as caregivers (20). The protector must support the individual, make sure they receive treatment, and ensure they do not harm themselves or others (120). This element, a corollary of a collectivist society par excellence, tends to limit, albeit partially, the role and liability of the psychiatrist.

There is a strong dichotomy regarding suicide. In some cases, there is the stigma surrounding it which minimizes both requests for help, while in others, it is idealized (20). In fact, although there has been an increase in cases of medical malpractice, the percentage of cases for the suicide of a patient is still very low as it's considered unpredictable (59).

In Japan, the gap appears wide between reality and the legislation. The interpretation of suicide has evolved, from an initial positive meaning to a personal problem, and more recently into a social problem. In addition to a possible diagnosis of a mental issue, emphasis is currently given, among other things, to work overload, poverty, parental burnout and caregiver overload, and social isolation (20).

The gap between Japanese culture and the Mental Health Law essentially consists in the dichotomy between collectivist society and individual autonomy. Furthermore, the idea of suicide as a positive moral act, as self-sacrificial, and as such, potentially honorable and virtuous, is sometimes idealized in a triumphalist way, still appears to be a partial fixture in Japanese culture. From this perspective, instead of being seen as a denial of the value of life, it takes on the meaning of the value of a moral duty towards others. The Japanese collectivist perspective is also influenced by Confucianism which subjugates individual desires to obedience and to loyalty to one's group (120).

#### 3.3.6 Korea

Article 10 of the Korean Constitution states that every citizen shall enjoy human dignity and the right to pursue happiness. Regarding the entire therapeutic process, Korean legislation has tended to give an important role to family members, allowing, for example, hospitalization based on an agreement between family members and psychiatrists in a context of tight control of the patient. A step forward was made with the Act for Prevention of Discrimination against Disabled Persons and the Protection of Their Rights (2007) which, among other things, obliges the family to protect the rights of the patient. In previous years, hospitalizations were overwhelmingly involuntary (91%) and very frequently due to an agreement between the family and psychiatrists. However, the provision of the act which provides for rehabilitation remains more declarative than effective and there is no focus on giving value to mental health care, which is still discriminated against in a judicial system that is more restorative than preventive (3).

The Mental Health Act of 2014 allows for involuntary treatment under extreme circumstances but overall underlines the importance of care over custodialism, and the defense of human rights as well as the duties of citizens. It emphasizes the role of family members as legal guardians to ensure the care and protection of rights of a mentally disturbed person, as well as to protect them from harming themselves or others (96).

Lawsuits against psychiatrists mainly concern hospitalized patients, with 66% decided in favor of the plaintiffs for negligence and violation of departmental management principles. Convictions following the death of the patient frequently involved negligence, violations in the management of the departments, insufficient provisions regarding emergency treatment, or delayed diagnosis or transfer. It would seem, however, that this legislation rarely entails the responsibility of the psychiatrist regarding outpatients who, among other things, represent a limited part of people with mental disorders given that psychiatrists represent 0.07 per 1000 compared to, for example, 0.17 in Canada and Brazil (4).

#### 3.3.7 Pakistan

Pakistan legislation is based on Islamic values and the fact that Islam considers suicide a sin is transposed into the legal concept of suicide, as, according to art. 309 of the penal code, it is considered a crime punishable with fines and/or prison. However, an indictment of attempted suicide is rare. By law, suicides and attempted suicides must be reported to the police, and often patients and their families prefer to go to private hospitals rather than to medical-legal centers to avoid mistreatment, embarrassment, and stigma. In rare cases in which mental illness is also diagnosed, the person who has attempted suicide then has access to treatment rather than punishment. The availability of treatment, however, is rather limited as in Pakistan there are only 343 psychiatrists and 478 psychologists for 200 million inhabitants (74).

The criteria for involuntary treatment are to save the patient's life, prevent a serious deterioration of their condition or alleviate suffering, or to prevent violent conduct of the patient towards themselves or others. Significantly, the request for involuntary treatment must be made by the wife, husband, or a close relative. If none of these figures are present, the reasons for their absence and the relationship of the applicant to the patient must be explained. A request may be sufficient to transport the patient to a psychiatric service and detain them against their consent for the period preceding the doctor's evaluation. The importance of the family is underlined by the possibility of requesting discharge with the codified commitment to take care of the patient and avoid risks for oneself or others (96). Therefore, the legal responsibility of the family is highlighted, which seems to significantly dilute that of the psychiatrist who often does not follow the patient.

#### 3.3.8 Thailand

Involuntary treatment is permitted when a patient is incapable of giving consent to protect or alleviate their mental disorder, or to prevent a person from harming themselves or others. For patients under 18 years old, or for those incapables of giving consent to the treatment, their spouse, ascendant, descendant, protector, curator, guardian or the person who takes care of them may give consent. Anyone who encounters a person whose behavior can make one reasonably presume a mental illness must notify the competent administrative office or the police who must take them to the nearest hospital (105). Stigma and poor resources (in 2013 there were 704 psychiatrists, i.e. 1.1 per 100,000 inhabitants despite the integration of mental health services into the public health system and a relative increase in psychiatric centers and hospitals) influences the resistance to seeking psychiatric care by people with mental illnesses and their relatives. Furthermore, despite adhering to the WHO guidelines in 2003, there is still a gap between the law and reality with an assumed underestimation of patient abuse. The role of healers, monasteries and religion remains important in the treatment of mental disorders, but so too does traditional Thai medicine, meditation and Chinese acupuncture (10). In this context, the psychiatrist's liability appears very limited.

### 3.4 Oceania

#### 3.4.1 Australia

In Australian (**Table 5**) forensic history there are no cases of criminal convictions against a psychiatrist for a patient's suicide. Civil lawsuits, however, are much more common. The civil code provides the legal framework for doctor liability. In Australia, due to federalism, the code varies locally based on the state; in any case, adherence to the guidelines established by the Royal Australian and New Zealand College of Psychiatrists is considered relevant (38).

**Table 5:** Oceania.

COUNTRY	LAW	CARE & LIABILITY	CRIMINAL SUICIDE	SUICIDE RISK CRITERION FOR INVOLUNTARY TREATMENT	MALPRACTICE
Australia	Civil Code, different for every state of the federation	Civil responsibility in prevention, according to the guidelines	Suicide not criminalized Euthanasia legal in some states.	Depending from the State, the psychiatrist can make a Temporary treatment order which a tribunal can transform in a Treatment order	Infrequent criminal liability, civil liability according to the duty of care (guidelines)
New Zealand	Accident Compensation Corporation (ACC), Health and Disability Commissioner (HDC)	Civil responsibility in prevention, according to the guidelines	Suicide not criminalized. Legal euthanasia. *Euthanasia for psychiatric patient allowed	Serious danger for the person or the others, seriously diminished capacity to take care of himself	No criminal liability, civil liability according to the duty of care (guidelines)

#### 3.4.2 New Zealand

Like Australia, civil liability is more emphasized than criminal liability in New Zealand. However, it should be noted that the presence of the Accident Compensation Corporation (ACC) Scheme limits the recurrence of lawsuits; in fact, every individual who is harmed automatically receives compensation (34). However, there is a Health and Disability Commissioner (HDC) who investigates complaints made against healthcare workers, and who, in addition to resolving disputes, can indicate the need to proceed with disciplinary sanctions; however, this rarely happens (78).

### 3.5 Europe

In Europe (**Table 6**), according to the literature as of 2015, there wasn't a high rate of litigation and convictions for malpractice in psychiatry (65). However, European legislation can attribute liability to the psychiatrist for the patient's suicide with important consequences for both patients and their doctors. Psychiatric malpractice suits have been increasing in recent years, leading also to an increase in the use of defensive medicine, which is intertwined with a certain perspective on mental illness. One of the main bases for psychiatric malpractice suits for cases of patient suicide seems to be a lack of hospitalization.

**Table 6:** Europe.

COUNTRY	LAW	CARE & LIABILITY	CRIMINAL SUICIDE	SUICIDE RISK CRITERION FOR INVOLUNTARY TREATMENT	MALPRACTICE
Austria	Responsabilities in prevention by psychiatrists	Responsabilities in prevention by psychiatrists	Suicide not criminalized Assiste suicide is not criminalized	Not included	Infrequent claims for penal liability, duty of care according to the guidelines
Belgium	Belgian Act of euthanasia	Ambiguity about psychiatric liability in preventing suicide	Suicide not criminalized Euthanasia permitted to psychiatric patients	Danger to themselves or others	Psychiatrists liability:mildly frequent claims also inherent in the ratification of euthanasia
Denmark	Mental Health Act 2010?	Responsabilities in prevention by psychiatrists	Suicide not criminalized	Danger to themselves or others	Psychiatrists liability:mildly frequent claims.
France	French criminal code, Article 121	Ambiguity about psychiatric liability in preventing suicidev	Suicide not criminalized	Need for urgent treatment and medical supervision	Psychiatrists liability:mildly frequent claims.
Germany	No specific legislation.	Responsabilities in prevention by psychiatrists	Suicide not criminalized	Danger to themselves or others	Psychiatrists liability:mildly frequent claims.
Greece	No specific legislation.	Responsabilities in prevention by psychiatrists	Suicide not criminalized	Danger to themselves or others	Psychiatrists liability:mildly frequent claims.
Ireland	Mental Health Act 2001	Responsabilities in prevention by psychiatrists	Suicide not criminalized	Danger to themselves or others	Psychiatrists liability:mildly frequent claims.
Italy	Basaglia Law 1978	Ambiguity about psychiatric liability in preventing suicide	Suicide not criminalized	Not included	Psychiatrists liability: frequent claims
The Netherlands	Compulsory Mental Health Care Act (CMHCA)	Responsabilities in prevention by psychiatrists	Suicide not criminalized Euthanasia permitted to psychiatric patients	Danger to oneself or others	Psychiatrists liability:mildly frequent claims also inherent in the ratification of euthanasia
Portugal	Decree nr 23/XV	Responsabilities in prevention by psychiatrists	Suicide not criminalized Euthanasia permitted to psychiatric patients	Danger to oneself or others, refusal for treatment	Psychiatrists liability: mildly frequent claims also inherent in the ratification of euthanasia
Scandinavia	Civil liability, regulated by Tort Laws	Civil liability in the decease or harm	Suicide not criminalized	Norway and Finland: significative danger for himself or the others Sweden: not setting specific criteria	Infrequent claims for penal liability, duty of care according to the guidelines
Spain	Organic law for the regulation of euthanasia	Responsabilities in prevention by psychiatrists	Suicide not criminalized Euthanasia permitted to psychiatric patients	Inability to take decision and care for oneself	Infrequent claims for penal liability, duty of care according to the guidelines
Switzerland	Swiss criminal code Dignity act	Ambiguity about psychiatric liability in preventing suicide	Suicide not criminalized Euthanasia permitted to psychiatric patients	Impossibility to provide the required treatment otherwise	Infrequent claims for penal liability, duty of care according to the guidelines
The United Kingdom	Mental Health Act 1983	Responsabilities in prevention by psychiatrists	Suicide not criminalized	Required detention for assessment; detention in the interest of health and safety of patiens and others.	Psychiatrists liability:mildly frequent claims.

In Europe there are several international documents which focus on Human Rights that are available as guidelines for state legislations, such as the Declaration of Hawaii from 1983, the Principles for the Protections of Persons with Mental Illness, the Ten Basic Principles for Mental Health Law distributed by the WHO, and WHO Mental Health and Human Rights (113). There is a slight difference in criteria for involuntary treatment among European countries. For example, in Italy and Spain there is no dangerousness criterion. Instead, they underline the need for a treatment criterion to prevent stigma (113).

#### 3.5.1 Austria

Austria had one of the most rigorous prohibition systems against suicide assistance in line with the European Court for Human Right underling that adequate measures to safeguard the lives of subjects under the jurisdiction of the state must be respected, even in the sphere of public health, and that the possibility of carrying out compulsory hospitalizations and the duty to ensure adequate protection for the patient is recognized as stemming from there. Additional to these positive obligations, the Court recognizes preventive protection measures that the state, and the treating psychiatrist, may be required to take to protect a subject from themselves (70). In 2020, changes are proposed that highlight the importance of the individual's right to self-determination: until 2022, article 78 of the Austrian Penal Code (ÖStGB) read: Anyone who induces others to kill themselves or helps them is punishable by imprisonment of six months to five years to punish. Since 2022, the word 'or helps them' are removed from the article, that now reads: Anyone who induces another to kill himself is punishable by imprisonment of six months to five years. Introducing, therefore, the possibility of assisted suicide

#### 3.5.2 Belgium

The law of 28 May 2002 authorized assisted suicide Belgium (57). The conditions for one to decide to end one's life are like those in other European countries: a voluntary, thoughtful, and repeated desire combined with significant suffering that cannot otherwise be mitigated (57).

Although Belgium is one of the European states most attentive to the right to euthanasia, according to a law issued in 2017, compulsory medical treatment is justified if the patient represents a danger to themselves or others (96). The difficulty then arises in identifying the boundary between the legitimacy of the psychiatric patient's desire for death and the responsibility of the psychiatrist, in the face of this situation, who has the burden of determining the individual's capacity for discernment. It is possible to deduce, therefore, that the most influential element on the psychiatrist's liability for the suicide of a patient is the way in which it occurs: through an impulsive action or through the expression of this desire to their doctor and the initiation of the final evaluative, decisional procedure in Belgium.

#### 3.5.3 Denmark

In 2020, the Compulsory Mental Health Care Act (CMHCA) was introduced in Denmark. The criteria for compulsory treatment (which in Denmark can only be decided by psychiatrists) include the dangerousness criterion (96).

The focus of the Mental Health Act (MHA) is the protection of the human and legal rights of patients (96). Traditionally, people with mental illnesses (excluding dementia and intellectual disabilities), regardless of type and severity are considered capable of deciding on medical treatments (19). In Denmark, the rate of convictions for malpractice suits by patients with mental disorders is lower than that for other patient groups. Furthermore, the acceptance rate of psychiatric patients' complaints is decidedly lower than that of somatic patients, perhaps also due to the greater difficulty of evaluating the success of preventive treatment in bipolar disorder (102).

The conditions for the provision of compensation are stricter than for other experienced specialists and are only implemented for injuries sustained during examination, treatment or similar conditions when the person acts below these standards and the suicide could have been avoided if those standards were followed. If the suicide or attempted suicide could not have been avoided, regardless of whether the treatment was in line with the expert specialist's standard or not, the complaint is rejected in pursuit of the no injury rule as the suicide cannot be considered a consequence of the treatment but rather a consequence of the patient's pathology (102). The implementation of preventive measures, such as concrete monitoring of the patient, is not always considered part of the treatment. However, when suicide is committed in hospital, a 2009 ruling of the Danish Supreme Court established that surveillance constituted part of the treatment during hospitalization (102).

#### 3.5.4 France

About the criminal liability of the psychiatrist, as with other legislation, the key element is the causal link between the doctor's incorrect behavior (due to imprudence, negligence, or insufficiency) and the damaging event that occurred. Article 121-3 para. 4 of the French criminal code provides that persons who did not directly cause the damage, but did not take adequate measures to avoid it, are criminally liable if it is established that they have committed [willful misconduct] or serious negligence that has placed others at a particularly high risk that could not be ignored (39). A certain legislative gap emerges in which a standardized reference to the good practice of the psychiatrist, and therefore of the suicide prevention, is missing since everything is reduced to the question of whether a normally prudent psychiatrist, who had worked on the same case, would have made the same decisions.

#### 3.5.5 Germany

An article by Henning Lorenz (2019) attempts to clarify aspects of third-person liability for suicide in German legislation following the decriminalization of suicide, even when the person's involvement was in the form of omission or incompetence. During the discussion, a definitive conclusion is not reached, thus highlighting the deficiency of German legislation regarding the topic of suicide, particularly in patients with suicidal tendencies. These patients may also have the right to self-determination similarly to patients in other medical contexts (61). At the same time, we find dangerousness towards others or the implementation of self-destructive attitudes in the criteria for the establishment of involuntary treatment (96).

#### 3.5.6 Greece

I will not administer to anyone, even if requested, a deadly drug, nor will I suggest such advice (32). With this phrase Hippocrates sanctioned the doctor's obligation to pursue life, in any circumstance and situation. Consistent with its history, Greece, to date, has not issued acts in favor of euthanasia, whether for physical or psychological problems. The psychiatrist is forced to intervene through compulsory health treatment when the patient shows criteria of social danger, if they lack the ability to objectively judge their own state of health and, in general in cases in which failure to hospitalize could lead to a worsening of the patient's state of disease (32).

#### 3.5.7 Ireland

In Ireland, psychiatrists are liable for preventing patient suicide, even by implementing coercive and custodial methods. In fact, the Mental Health Act of 2001, although representing progress compared to previous legislation, does not sufficiently eliminate the paternalistic and custodial element with respect to mental illness (28). There currently seems to be no studies defining psychiatrist liability regarding suicide prevention.

#### 3.5.8 Italy

In Italy, the question of a psychiatrist's liability has passed through three phases: custody, indulgence, and assigning liability. According to the 1904 Italian Act on Provisions on Asylums and Insane Persons (Custody and Care of the Alienated) (51), the psychiatrist's responsibility was custodial and it was their duty to defend society from the mentally ill who were considered dangerous and/or actors of public scandal. In this context, professional liability was that of custody and non-custody and on failure to report mental illness or serious, potentially dangerous infirmities. During the indulgence phase, the Basaglia Law of 1978 (also known as the 180 Law) revolutionized the psychiatric system in Italy (52), referencing Article 32 of the Italian Constitution which states that the Republic protects health as a fundamental right of the individual and the community and guarantees free treatment to the indigent. No one can be forced to undergo a specific health treatment except by law (62). There were some acquittals in the 1990s due to the fact that there was no obligation to require involuntary treatment, a measure to be adopted with discretion and in any case not foreseen by the legislation for the prevention of suicide. Differently, in the present accountability phase, the position of guarantee gradually risks returning to the custodial obligation whereby the danger to oneself and others is considered as a psychic alteration such as to require urgent therapeutic interventions. The custodial obligation, also required in a voluntary hospitalization regime, appealing to a consensual custody regime, has been highlighted in some convictions but is also contained in acquittals (62).

With sentence nr. 4391 of 22/11/2011, the Court of Cassation underlined how a treatment method that does not include segregation and isolation, and which therefore respects the dignity of the patient, cannot completely eliminate the danger of suicide, thus outlining permitted risk. The presence of this margin of risk does not relieve the psychiatrist of their responsibilities. As reiterated by sentence nr. 24138 of 25 May 2022 of the Fourth Criminal Section of the Court of Cassation, the psychiatrist holds a position of guarantee which includes an obligation to control and protect patients, aimed at preventing the risk of them committing harmful acts and behaviors prejudicial to themselves. In this way, if the risk of suicidal actions by a patient is defined as insurmountable, the need for a form of control is highlighted, especially during hospitalization. There is a recognizable difficulty in balancing the protection of a patient's freedom and the need to guarantee care for the patient and the current interpretation of the guarantee position frequently has a custodial overtone (62).

The link between the guarantee position, preventive obligations, and precautionary rules has been investigated in light of the permitted risk and the scope of the doctor's duties (27). The position of guarantee theoretically implies an obligation to control and protect the patient from behaviors that can lead to suicide. The author doubts that the psychiatrist should be the guarantor not only of the patient's health but also of their physical safety and the safety of others. The transformation of the wording in the 180 law from dangerous subject into patient and co-protagonist of the therapeutic relationship delimits the position of guarantee which should not include the prevention of crimes by the patient, outside the application spectrum of Article 32 of the Italian Constitution (27) The Court of Cassation has confirmed the existence of the crime of abandonment of an incompetent person when it is characterized by the nature of a crime of concrete danger, related to the type of incompetence. The crime of the abandonment of minors or incompetent persons can be configured with the failure to propose involuntary treatment (62).

#### 3.5.9 The Netherlands

The Netherlands became part of the CRPD (United Nation Convention on the Rights of Persons with Disabilities) in 2016, and in 2020, it promulgated the Compulsory Mental Health Care Act (CMHCA), which authorizes involuntary treatment under exceptional circumstances (5).

Two types of involuntary treatment are provided by law: short-term and prolonged hospitalization, contain the criterion of danger to oneself or others (96). Euthanasia for terminal or non-terminal illness, including psychiatric disorders, has been permitted in Belgium and the Netherlands for at least 20 years. Although, there has been an increase in euthanasia, the percentage of cases with psychiatric diagnoses has remained stable at 1-2%. The criteria for euthanasia are: a well-considered, voluntary, and repeated request by a legally competent adult; a pathology with no prospect of improvement; constant, unbearable suffering that cannot be relieved; and consultation of two independent doctors including a psychiatrist as well as subsequent follow-up (29).

Medically assisted suicide has increased for psychiatric patients from 2-5 cases in 1997 to 56 in 2015 (31). However, there are many doubts regarding the identification of decision-making capacity, and the use of medically assisted suicide is controversial. Establishing whether the desire to die is a pathologically determined choice or a rational one is difficult also because the decision-making capacity of the patient tends to fluctuate and is often diminished. Capacity is presumed in the Netherlands, in more than half of the cases, based primarily on the persistence of the choice despite the presence of disorders that increase the risk of incapacity. In fact, a low threshold is highlighted despite the great prudence recommended by the RTE euthanasia review committee (Regionale Toetsingscommissies Euthanasia) (31).

There are various hypotheses regarding the differences between suicide and assisted suicide or euthanasia. If some clinicians from the American Association of Suicidology see greater impulsiveness in the former and a planned and appropriately considered act in the latter, others do not embrace these differences, also because the impulsiveness seems to relate more to attempted suicides (75).

In this relatively liberal context, there is softened psychiatrist liability regarding suicide, which is also inherent in the ratification of euthanasia or medically assisted suicide. In fact, there are sometimes lawsuits by relatives against doctors who have approved euthanasia requests for psychiatric patients. These protests have led to more restrictive legislation in Belgium for the euthanasia of these patients (29).

#### 3.5.10 Portugal

In 2023, Portugal joined the countries that have legislated regarding the possibility of medically assisted suicide. In decree nr. 23/XV it is declared that medically assisted death is not condemnable when it occurs by the decision of an adult person, whose will be current and reiterated, serious, free, and informed, in a situation of great intensity of suffering, with a definitive injury of extreme severity or a serious and incurable disease, when practiced or assisted by healthcare professionals (85). According to Article 2 (law nr. 22/2023), great intensity of suffering means physical, psychological, and/or spiritual suffering resulting from a serious and incurable disease or permanent injury of extreme severity, of great intensity, persistent, continuous, or permanent, and considered intolerable by the person themselves (86). Among the valid criteria for requesting involuntary treatment, Portugal includes danger to oneself or others, together with refusal of treatment and the consequent liability for psychiatrists (96).

#### 3.5.11 Scandinavia

In Norway, Sweden and Finland, there are no reports of criminal cases against psychiatric doctors for patient suicide, nor specific legislation regarding the issue. Cases of negligence, incompetence or malpractice, from a civil point of view, are covered under a law on civil liability (Tort Law), different for each state, but with common characteristics such as the demonstration of the damage suffered being the responsibility of the plaintiff (55, 48, 89). These laws also apply to cases of suicide in hospital settings. It seems reasonable to think that this situation is linked on the one hand to the healthcare system based essentially on insurance, and on the other to a vision of suicide as a free choice of the individual: in all three countries it is in fact a non-prosecutable act. The connotation of suicide as a free choice fits into the context of these countries.

#### 3.5.12 Spain

On 25 June 2021, Spain officially produced legislation regarding euthanasia with the aim of legitimizing the desire to end one's life when specific conditions exist. Specifically, in the presence of a serious, chronic, and highly debilitating illness, and with the competence to decide. Although the state has recognized the right to euthanasia, a 2022 article by Sorzano stresses the need for an update of the guidelines for suicide prevention and underlines the need to identify and unify the criteria for identifying patients at risk (103). To support the psychiatrist in evaluating who would be fully capable of making such a decision, various models have been proposed based on a cognitive evaluation (7). Other authors highlight the importance of studying the patient's insight or the clinical relationship (103).

It is worth considering that Spain does not include a dangerousness criterion among the criteria for the implementation of involuntary treatment, instead relying on the presence of a mental disorder which causes significant difficulties in taking care of and making adequate choices for oneself (96). The psychiatrist's task therefore becomes to determine whether the person can decide for themselves. If the patient wishes to end his own life, the decision becomes whether to legitimize the suicide or request involuntary treatment, without a tool that can really protect the doctor by objectively demonstrating the legitimacy of the patient's desire to commit suicide.

In Spain, up to 2015, legal suits against psychiatrists were few and generally had little consequence on the accused (65).

#### 3.5.13 Switzerland

In 1941, Switzerland became the first country to legally permit assisted suicide. In the last twenty years, the discussion has reopened regarding the need to legislate euthanasia to update the terms and conditions, but in June 2011, with an official statement, the federal office of Justice explicitly renounced regulating organized assistance to suicide in criminal law (25).

A 2021 Swiss study by Michel Sabe aimed to examine the difficulty in offering a proper level of assistance to prevent a suicidal act and provide a framework for the management of these cases, trying to clarify one of the most complex and unclear aspects of forensic medicine (94).

In Switzerland, the practice of lay Right-To-Die societies (RTDS) organizing assisted suicide is tolerated by the state (90). Patients are generally reached by members of the societies. The role of doctors is limited to prescribing the lethal dose and evaluating the patient's decision-making capacity and whether the pathology causes intolerable suffering and if treatments would be ineffective. Such tolerance for assisted suicide does not preclude the obligation to prevent suicide. About suicide prevention, the legislation appears scant to determine in which situations doctors are obliged to act. In the revised Swiss Criminal Code, the only requirement for assisted suicide remains decision-making capacity (90). The constant involvement of doctors clashes with the dominant role of non-medical RTDS members (90).

In line with the legality of euthanasia, Switzerland does not include danger to the patient's life in the criteria for involuntary treatment, thereby making euthanasia a possibility also for psychiatric patients (96).

#### 3.5.14 The United Kingdom

Article 2 of the European Convention on Human Right states: Everyone's right to life shall be protected by law. No one shall be deprived of his life intentionally save in the execution of a sentence of a court following his conviction of a crime for which this penalty is provided by law (82).

The English Supreme Court unanimously determined that healthcare professionals operating in the mental health field have an operational obligation to protect patients from suicide. Although the United Kingdom has progressively transposed the European Convention on Human Rights into their legislation, involuntary treatments continue to be carried out on people with intact decision-making, contrary to what happens for physical diseases (123).

A final report from the Royal College of Psychiatrists identifies which pharmacological treatments are correlated with a reduction in the incidence of suicide. However, the risk assessment tools which were used in interventions have provided insufficient evidence to demonstrate their effectiveness (93). In this way, the tool of pharmacological therapy is favored by psychiatrists, and the risk of an unnecessary prescription, motivated only by defensive medicine, is increased.

## 4 Discussion

The ethical and legal aspects of patient suicide are multifaceted and peculiar to various parts of the world as there is no universal perspective of liability and the context of relatively scarce resources, stigma, and low valuation of patients' rights effectively dilutes the liability of psychiatrists (59). The obligations include the appropriateness of the diagnosis and therapy, as well as the duty of continuity of care. Some legislations specify the liability for ensuring the transition to another therapist. In fact, abandonment of a patient represents a frequent complaint (27). Moreover, the lack of communication to relatives of the risk of potential suicide (a delicate topic for both legal and relational implications) could implicitly fall within the context of abandonment. In this regard, the difference between a suicide carried out in a state of abandonment – either due to scarce state resources or due to the specific situation – and a suicide carried out in a care context, is noteworthy.

Considering this, the psychiatrist's liability regarding patient suicide partially, but significantly, overlaps with the observance of the criteria for compulsory treatment in which the so-called dangerousness criterion is present in most legislation worldwide. However, some countries, like Italy and Spain, do not include that criterion. In fact, the 1978 reform (180 Law) in Italy eliminated the obligation of custody and social defense, focusing instead on an obligation of care as the fundamental requirement in the presence of a mental disorder. An ambiguity in the Italian legal system concerns the situation that, although there is no such criterion, in the event of a suicide, the psychiatrist can be accused of not having carried out a needed involuntary commitment. If one part of the jurisprudence considers involuntary commitment illegitimate only to neutralize social danger in the absence of therapeutic necessity, on the other hand, the sentences presuppose that the psychiatrist must prevent all negative consequences that mental suffering can induce. This situation can result in therapeutic avoidance or abandonment and defensive medicine aimed at the social and personal defense of the psychiatrist. The voluntary assumption of a certain risk by a capable and informed patient who refuses treatment, unlike a patient incapable of assuming the risk, should exonerate the psychiatrist who must respect the patient's freedom of self-determination (27). In this regard, many legislations emphasize the connection between a psychiatrist's liability and a patient's inability to legally consent. There is often a contrast between the precautionary attitude and the therapeutic goal both for the regressive effect and for the effect of inducing passivity that a hospitalization can cause and for the reverberation that a hospitalization, even more so if involuntary, has on the doctor-patient relationship. Another legal problem may stem from the non-hospitalization of a patient, even when agreed to by the patient. However, hospitalization is not neutral as it can favor a regressive aspect, which often determines a negative impact on the possibility of treatment, especially when the patient also follows psychoanalytic psychotherapy.

Appel claims that to protect themselves from liability, psychiatrists have to engage in over-inclusive approaches to hospitalization: denying freedom to 99 patients at risk who will not kill themselves, so to speak, to protect the one patient who will do so. He notes that while it may offer protection to the one, the negative consequences for the other 99 patients should not be underestimated. He also states that such treatment may have positive short-term outcomes but could lead to an increase in suicides if the hospitalizations cause patients to avoid future care. Another consequence noted is that psychiatrists may try to avoid patients with suicidal risk (6).

In clinical practice there is often a tendency to act with so-called defensive medicine, leading psychiatrists to react immediately with pharmacological therapies and/or hospitalizations in situations that do not always involve a concrete risk, which can damage the main figure to be protected, i.e., the patient. Additionally, a type of paradox appears in some countries in which the psychiatrist seems to have a duty to restrict a patient's freedom, while also being legally restricted from doing so (122).

One may wonder whether the guarantee position required by the psychiatrist should concern the preservation of life, or the pursuit of the greatest possible happiness or, to be less naive, the greatest possible reduction of suffering. The difference between impulsive suicide and an enduring desire to die, is interesting and should be addressed in future study. Furthermore, even from the perspective of prevention, it can be noted that environments which are too restrictive or too permissive can be equally destructive. The predictability of suicide is expressed as a gradation of risk and this difficult to identify gradation can take caution to extremes, thus hindering the therapeutic process. In addition to quantity, the quality of the risk must also be considered.

The responsibility of psychiatrists in cases of suicide remains a complex issue, because of the variability across jurisdictions. Ambiguities in legislation leave space for interpretation regarding the psychiatrists' liability, especially in cases involving voluntary admitted patients (14). This complexity is compounded by ethical considerations that have to respect the patient's autonomy with the necessity to prevent harm, especially in countries with limited resources (14).

Many best practices for suicide prevention have been written in order to guide psychiatrists in managing these challenges. Risk assessment plays a critical role, starting from factors such as psychiatric diagnoses, number hospitalization, and histories of suicide attempts (43). Particular attention is required during high-risk periods, such as the first month following discharge from inpatient care or in difficult periods in the lifetime. Transitioning patients from inpatient to outpatient care is also important, necessitating clear communication, territorial structure and care strategies, and the involvement of family and community supports in safety planning. Trauma-informed care, which recognizes the impact of adverse experiences, has also been highlighted as essential for mitigating suicide risks (43).

Ethical and clinical issues include summarizing the tension between preventing suicide and respecting patients' autonomy (14).

The World Health Organization's guidance suggest to integrate mental health and human rights perspectives into practice. This involves promoting universal access to high-quality mental health care, building capacity among healthcare providers, and adhering to non-coercive interventions, remembering human rights standards (115).

The WHO underlined the need of elimination of discrimination against people with mental health conditions, calls for laws that promote access to care and support in the least restrictive environments possible, respecting patient autonomy and ensure informed consent (115).

We suggest that global guidelines are needed to establish psychiatrists' responsibilities and to expand on collaborative care models that involve peer-to-peer support, family engagement, and community resources (115).

What emerges from this study is the gray area of psychiatric patient suicide. Is it possible to make the psychiatrist liable for an unmanageable illness? What are the correct guidelines? When the possibility of coercion is no longer valid to avoid suicide and when does the right to self-determination begin for the psychiatric patient?
